# Pressure-Retaining Sampler and High-Pressure Systems to Study Deep-Sea Microbes Under *in situ* Conditions

**DOI:** 10.3389/fmicb.2019.00453

**Published:** 2019-04-09

**Authors:** Marc Garel, Patricia Bonin, Séverine Martini, Sophie Guasco, Marie Roumagnac, Nagib Bhairy, Fabrice Armougom, Christian Tamburini

**Affiliations:** ^1^Aix Marseille Univ., Université de Toulon, CNRS, IRD, MIO UM 110, Marseille, France; ^2^Sorbonne Université, CNRS, Laboratoire d'Océanographie de Villefranche, LOV, Villefranche-sur-Mer, France

**Keywords:** pressure-retaining sampler, deep sea, *in situ* sampling, prokaryotic activities, prokaryotic diversity

## Abstract

The pelagic realm of the dark ocean is characterized by high hydrostatic pressure, low temperature, high-inorganic nutrients, and low organic carbon concentrations. Measurements of metabolic activities of bathypelagic bacteria are often underestimated due to the technological limitations in recovering samples and maintaining them under *in situ* environmental conditions. Moreover, most of the pressure-retaining samplers, developed by a number of different labs, able to maintain seawater samples at *in situ* pressure during recovery have remained at the prototype stage, and therefore not available to the scientific community. In this paper, we will describe a ready-to-use pressure-retaining sampler, which can be adapted to use on a CTD-carousel sampler. As well as being able to recover samples under *in situ* high pressure (up to 60 MPa) we propose a sample processing in equi-pressure mode. Using a piloted pressure generator, we present how to perform sub-sampling and transfer of samples in equi-pressure mode to obtain replicates and perform hyperbaric experiments safely and efficiently (with <2% pressure variability). As proof of concept, we describe a field application (prokaryotic activity measurements and incubation experiment) with samples collected at 3,000m-depth in the Mediterranean Sea. Sampling, sub-sampling, transfer, and incubations were performed under *in situ* high pressure conditions and compared to those performed following decompression and incubation at atmospheric pressure. Three successive incubations were made for each condition using direct dissolved-oxygen concentration measurements to determine the incubation times. Subsamples were collected at the end of each incubation to monitor the prokaryotic diversity, using 16S-rDNA/rRNA high-throughput sequencing. Our results demonstrated that oxygen consumption by prokaryotes is always higher under *in situ* conditions than after decompression and incubation at atmospheric pressure. In addition, over time, the variations in the prokaryotic community composition and structure are seen to be driven by the different experimental conditions. Finally, within samples maintained under *in situ* high pressure conditions, the active (16S rRNA) prokaryotic community was dominated by sequences affiliated with rare families containing piezophilic isolates, such as Oceanospirillaceae or Colwelliaceae. These results demonstrate the biological importance of maintaining *in situ* conditions during and after sampling in deep-sea environments.

## Introduction

Only 5% of the ocean has been explored using remote instruments and <0.01% has been sampled and studied (Ramirez-Llodra et al., 2010). Despite the deep sea, being the largest ecosystem of the ocean (mean depth of about 3,500 m, i.e., 35 MPa), with the greatest reservoir of microbes (Whitman et al., 1998), it still hasn't been sampled or studied adequately under *in situ* conditions. This major issue is mainly the consequence of technical limitations that both restrict access to this environment and make sampling under *in situ* pressure conditions difficult.

Microorganisms living in the deep-sea are subjected to well-known conditions: high hydrostatic pressure, low temperature, high concentrations of inorganic matter, and low organic carbon content. Pressure-adapted microorganisms have been isolated from many deep-sea sites and are defined as piezophiles when their optimum growth occurs at high hydrostatic pressure. Using laboratory pressure vessels, the characteristics of piezophiles have been described, including membrane properties, motility, nutrient transport and DNA replication and translation under elevated hydrostatic pressure (see e.g., Bartlett et al., 2007; Lauro and Bartlett, 2008).

In deep-sea environments, microorganisms represent the main players in the biological carbon pump by consuming and mineralizing organic matter as it sinks down the water column (Cho and Azam, 1988; Aristegui et al., 2009). However, one of the main biases in deep-sea metabolic activity estimations is that, most of the time, they are measured at atmospheric pressure following the decompression of the water sample. As a result of decompression, significant shifts in prokaryotic activity (Tamburini et al., 2013; Wannicke et al., 2015), community composition (La Cono et al., 2009, 2015), and gene expression (Edgcomb et al., 2016) have been identified. In a previous review (Tamburini et al., 2013), we considered the hydrostatic pressure effects on deep-sea natural communities. Analysis of datasets in the literature, show that under stratified conditions, deep-sea communities are adapted to *in situ* conditions of high pressure, low temperature, and low organic matter. Measurements from decompressed samples that are incubated at atmospheric pressure thus underestimate *in situ* activities. Exceptions that can lead to overestimations can be attributed to deep sea mixing events, large influxes of surface particles, or the provision of excessive organic matter during experimentation (Tamburini et al., 2013).

In order to study the deep-sea environment, it is necessary to perform *in situ* measurements or continuously maintain the hydrostatic pressure at *in situ* temperature from deep-sea water sampling through to the transfer and measurement of microorganism activity and community composition. Over the last 40 years, pressure-retaining sampler prototypes have been developed by several laboratories using different approaches, but none are available to the scientific community without local engineering expertise or high-pressure system facilities. For the oldest systems refer to Bianchi et al. (1999), Jannasch et al. (1973), Tabor and Colwell (1976) or for an historical review of microbial pressure-retaining samplers see Tamburini (2006). Recently, new pressure-retaining samplers have been locally developed [without being exhaustive NIOZ, the Netherlands; JAMSTEC, Japan—(Kim and Kato, 2010); WHOI, USA—(McNichol et al., 2016); SCRIPPS, USA—(Peoples et al., 2019)].

Here we describe a ready-to-use pressure-retaining sampler capable of collecting and maintaining samples under *in situ* pressure conditions (up to 60 MPa) during sampling and the ascent from ocean depth. Along with a complete high-pressure setup, we show how to perform experiments, including replicates, transfer, and subsampling, without decompression. Technical specifications and proof of concept are described with examples of data including oxygen concentration and prokaryotic diversity from incubation experiments. These latter were carried out from a deep natural prokaryotic community collected at 3,000 m-depth in Mediterranean Sea, a first series being maintained under *in situ* pressure compared to another series incubated at atmospheric pressure.

## Materials and Methods

### High-Pressure Bottles (HPBs)

Two kinds of High-Pressure Bottles (HPBs) are available, one in stainless-steel coated with PEEK (Poly-Ether-Ether-Ketone) described in Bianchi et al. (1999) and Tamburini et al. (2003), and one in titanium (described below). Different volumes can also be used: 500 mL HPBs used for sampling and 50 mL HPBs used for incubation.

Titanium alloy is widely used in oceanography instrumentation as it meets corrosion constraints linked to biofouling and prolonged immersion at great depths, or those seen in hydrothermal vents such as extreme temperatures and pH. Titanium, in particular TA6V ELI grade 5 used in the medical field, has several interesting mechanical properties when working at high-pressure. The tear resistance of TA6V is 3 times higher than 316L and the elastic limit of TA6V is 4 times higher than 316L. Densities are 4.33 g cm^−3^ and 8 g cm^−3^, respectively for TA6V ELI and 316L. Such differences facilitate manual handling. In addition, TA6V ELI, like other titanium, is resistant to corrosion, specifically to pitting and crevice corrosion.

**Figure 1:**
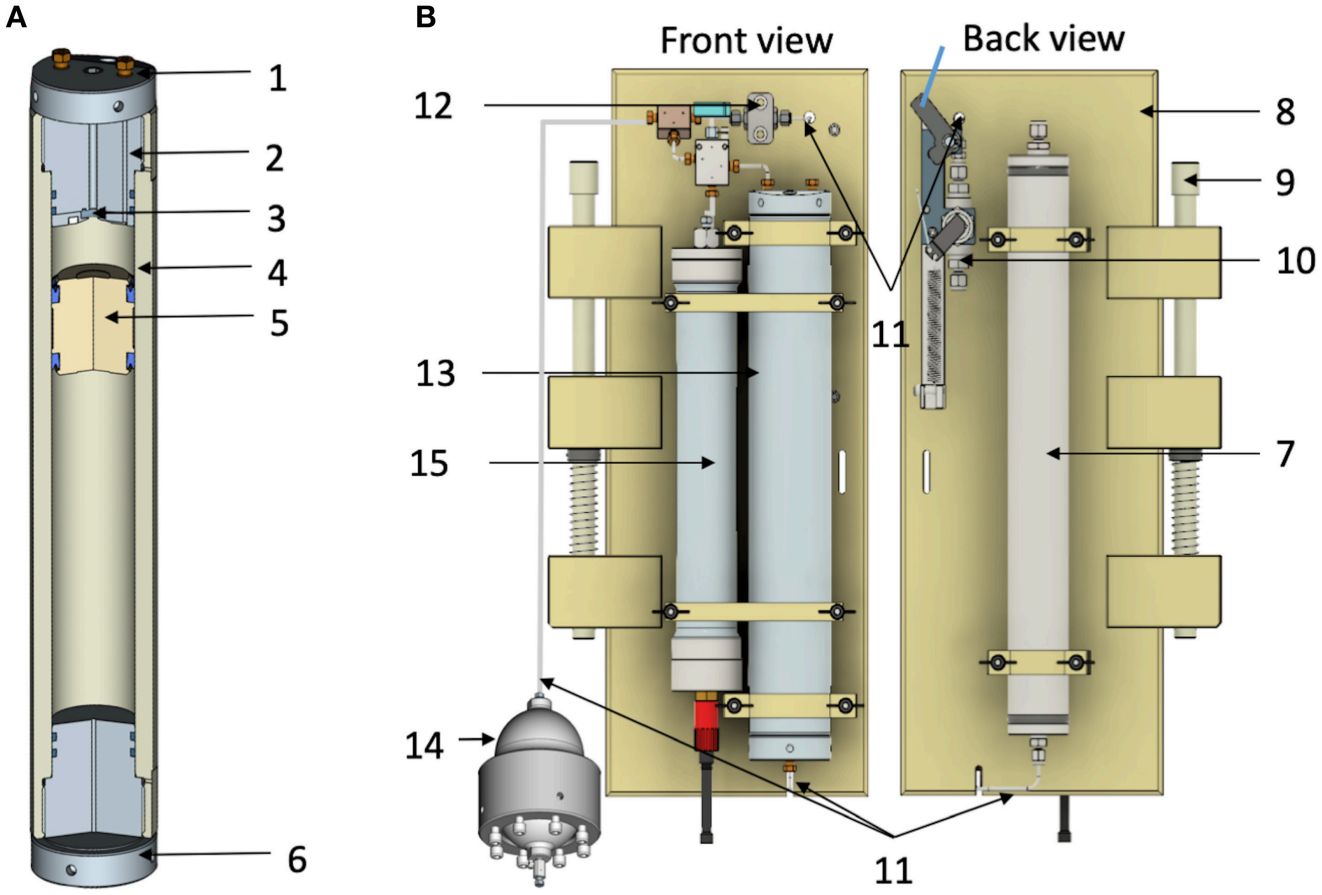
**(A)** Cross section of the Titanium TA6V 500 mL High Pressure Bottle (HPBs). **(B)** Schematic drawing of the front and back views of the High-Pressure Sample Unit (HPSU). 1. Top end-cap devoted to oxygen measurement; 2. 1/8″ (3.2 mm O.D.) Inlet flowthrough; 3. Sapphire window; 4. Main core; 5. PEEK floating-piston with lip seal; 6. Bottom end-cap. In the part, above floating-piston seawater sample is in contact with sapphire window (see details in the text). The part below the floating-piston contains sterile milliQ water which serve as hydraulic brakes when passing through the thin catheter into the exhaust tank (7); 8. Polypropylene main frame; 9. Push rod for attachment to a rosette system. When the (10) inlet-valve is opened by magnetically activated lanyard release, the seawater enters the hydraulic circuit through a 1/8″ (3.2 mm O.D.) stainless-steel tube (11), via one check valve (12), to fill in the HPB (13), the pressure accumulator (14), and the aero-hydraulic pressure sensor (15).

Based on our previous experience expertise, we built a new generation of HPBs in Titanium TA6V ELI grade 5 for medical use (2686-0000, Top Industrie SAS, https://www.top-industrie.com) avoiding the use of PEEK coating which is an expensive consumable. These bottles are similar in principle to those made in 316L stainless-steel with PEEK coating described by Bianchi et al. (1999) and Tamburini et al. (2003) (Figure 1A). An important additional improvement was made using a top-end cap devoted to optical measurements. Ti-HPBs are 500 mL Titanium TA6V ELI cylinders with PEEK floating-pistons fitted with two polyurethane seals as they are more suitable for longitudinal friction. Maximum operating pressure and operating temperatures are 60 MPa and 50°C. The screw top end-cap (in titanium TA6V ELI) fitted with two O-rings is composed of two parts (see details in Supplementary Image 1). Main part with 4 pipes, 2 screwed pipe connections 1/8″ (3.2 mm O.D.) for sample pathway, 1 blind pipe for PT100 sensor with a diameter of 6 mm and length 60 mm and 1 pipe for the optic fiber with a diameter 5 mm and a length of 82 mm. The second part is 4 mm thick, screwed into the main part with three titanium grade 5 screws to fix a sapphire window for optical measurements. The bottom end-cap (in titanium TA6V ELI) has only one connection (see in the text below).

#### Cleaning Procedure

HPBs are washed with pressurized milliQ water using a pre-cleaned stainless-steel pressurized container. For washing HPBs, pressurized milliQ water moves the floating-piston in two ways sequentially. This backward and forward movement is repeated three times and the floating-piston is placed at the top of the HPB. Hence, the part below the floating-piston is fully filled with milliQ water. Finally, HPBs were sterilized in a pressure-sealed unit (for 20 min at 110°C). A more thorough cleaning procedure can also be used as described in Tamburini et al. (2009b) depending on the application and before starting the cruise.

### High-Pressure Sampler Unit (HPSU)

To perform deep-water sampling with maintained elevated pressure, both 500 mL HPBs, described previously, are fitted onto a specific frame, designated the high-pressure sampler unit (HPSU, described in Figure 1B). The HPSU can be fitted onto a CTD carousel beside the Niskin bottles (using the same attachment push-rod system), widely used in oceanography but providing decompressed deep-water samples.

To make it accessible to potential future users, we have standardized the manufacturing of the HPSU in collaboration with Top Industrie SAS (Vaux-le-Penil, France). The HPB is fitted by two flanges onto a polypropylene frame (287 × 600 mm) and connected to the top-part at the inlet-valve (see Supplementary Image 2 for details) with a check-valve and to the bottom-part on the exhaust tank (a stainless-steel tank, 480 mm total length, 55 mm O.D.). The Supplementary Image 2 shows the schematic drawing of the quarter-turn inlet-valve, which is set to closed position, thanks to a nylon wire (under tension using a strong spring) on the corresponding CTD-carousel trigger until fired for sampling. Moreover, each HPB is linked to the hydraulic part of autonomous data-logger pressure sensors (2903-0000, Top Industrie SAS), these are independent of the CTD carousel and gives quality control of samples before processing. HPBs are connected to an aero-hydraulic pressure accumulator (2820-1300, Top Industrie SAS) to counterbalance drops in pressure (see the description in Bianchi et al., 1999).

### High-Pressure Sampling

**Figure 2:**
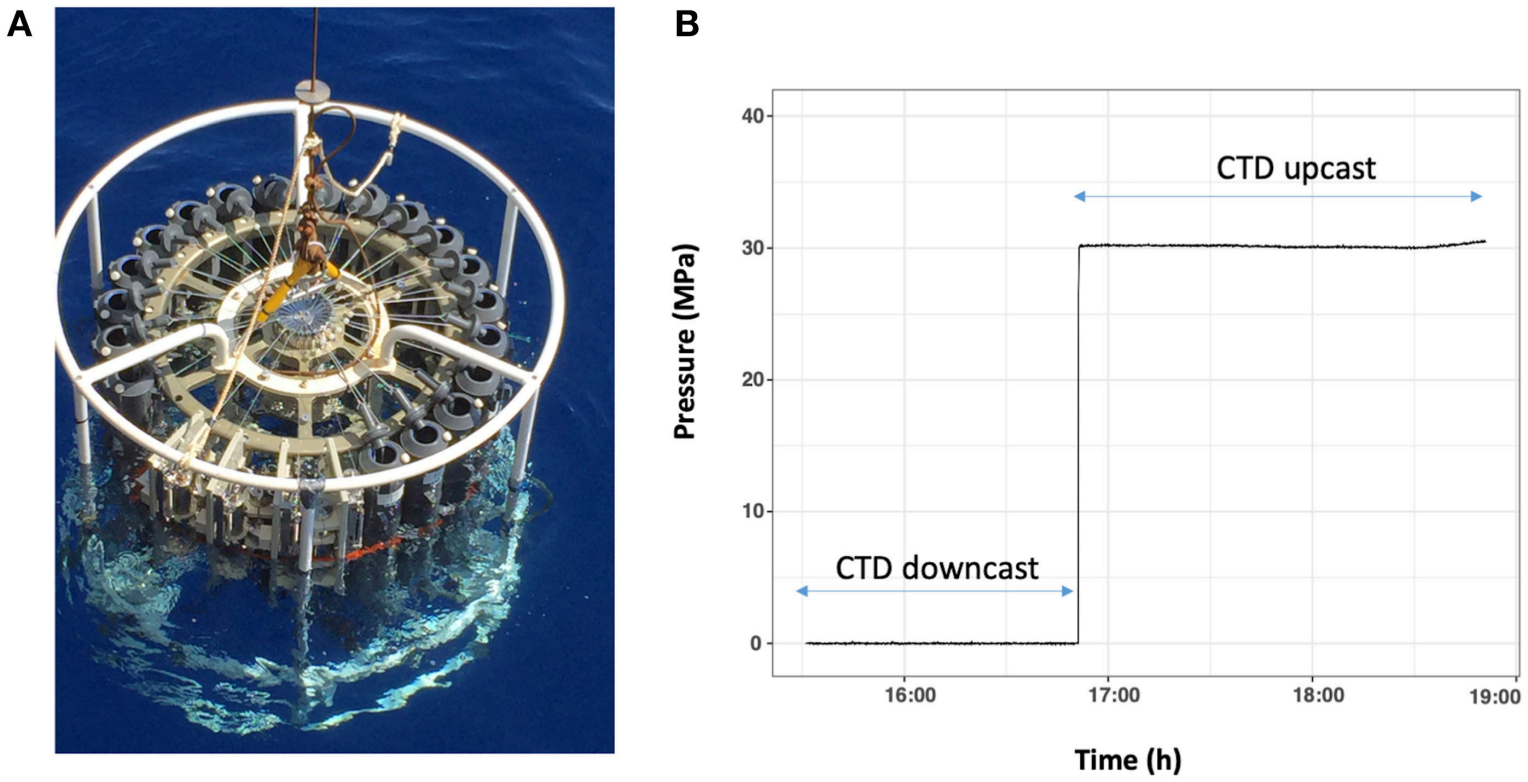
**(A)** Photograph of four HPSUs mounted on a rosette system. In this example, four HPSUs are taking the place of 5 Niskin-bottles. **(B)** Plot of recorded data by pressure sensor during sampling. Pressure sensor begins to record data when inlet-valve of frame is open. Data is recorded during all the casts and stored in data logger. Recorded data are available on board to check quality of collected samples.

For sampling, the HPSU is fitted to a CTD-carousel. The photograph in Figure 2A shows four HPSUs mounted on a 24-bottle CTD-carousel. At the desired depth, the CTD carousel trigger is fired, freeing a spring to open the inlet-valve with a quarter turn. The seawater enters the hydraulic circuit through a 1/8″ (3.2 mm O.D.) stainless-steel tube, via one check-valve, filling the HPB, the pressure accumulator and the pressure sensor. As the seawater enters the HPB, the floating-piston moves and flushes out the milliQ water below the floating-piston into the exhaust tank through a stainless-steel-tube (1/51″ I.D.−0.5 mm I.D.). This ensures a continuous and slow flow rate during the downward movement of the floating-piston and prevents damage to the floating-piston as the HPB fills up with seawater. Seawater sampling ends when the floating-piston sits on the bottom end-cap (Figure 1B and animated drawing in Supplementary Video 1). The check-valve means there is no need for a closing valve or an additional trigger. Once the inlet-valve is triggered to open, the sampling time is estimated to be around 10 min to guarantee full-filling and equilibrium with the pressure accumulator.

Once the HPSU is back on board, the master pressure sensor is connected to the computer to check the monitored pressure recorded during sampling and to determine its quality (pressure variability). It can be observed that the hydrostatic pressure is maintained at ± 5% during the up-cast of the carousel. Once on board the ship, HPSUs are recovered and transferred immediately into our mobile MIO-HPLab container (Supplementary Image 3). HPSUs are then placed in the *in situ* temperature water baths before being processed to offset the slight increase in temperature during up-cast and manual handling on deck. An example on Figure 2B shows a sample collected at a depth of 3,000 m, it can be observed that the hydrostatic pressure is maintained throughout the ascent without pressure variation.

### Transfer and Subsampling in Equi-Pressure Mode

An important improvement was made in transferring the main sample and several sub-samples (obtained with the HPSU) under an equi-pressure mode maintaining at all times the high hydrostatic pressure, using a piloted pressure generator (PPG). This procedure can be used to obtain replicates of the same activity measurement.

#### Piloted Pressure Generator

The piloted pressure generator is a PHMP 600-600 (2493-0000, Top Industrie SA). It consists of a TA6V reservoir with a maximum operating pressure of 60 MPa, a useful capacity volume of 600 mL and a maximum flow rate of 50 mL min^−1^. The PMHP 600-600 is filled with milliQ water that is compressed by a piston regulated by a brushless motor Servomotor Compax 3. This enables us to work with hydrostatic pressure safely. The PMHP 600-600 operates in three modes: pressure, volume and flow. The draining and filling/pressure input is controlled by two 1/8″ electro-valves piloted by Labview software (National Instruments™).

#### Protocol to Transfer and Subsample Under Equi-Pressure Mode

**Figure 3:**
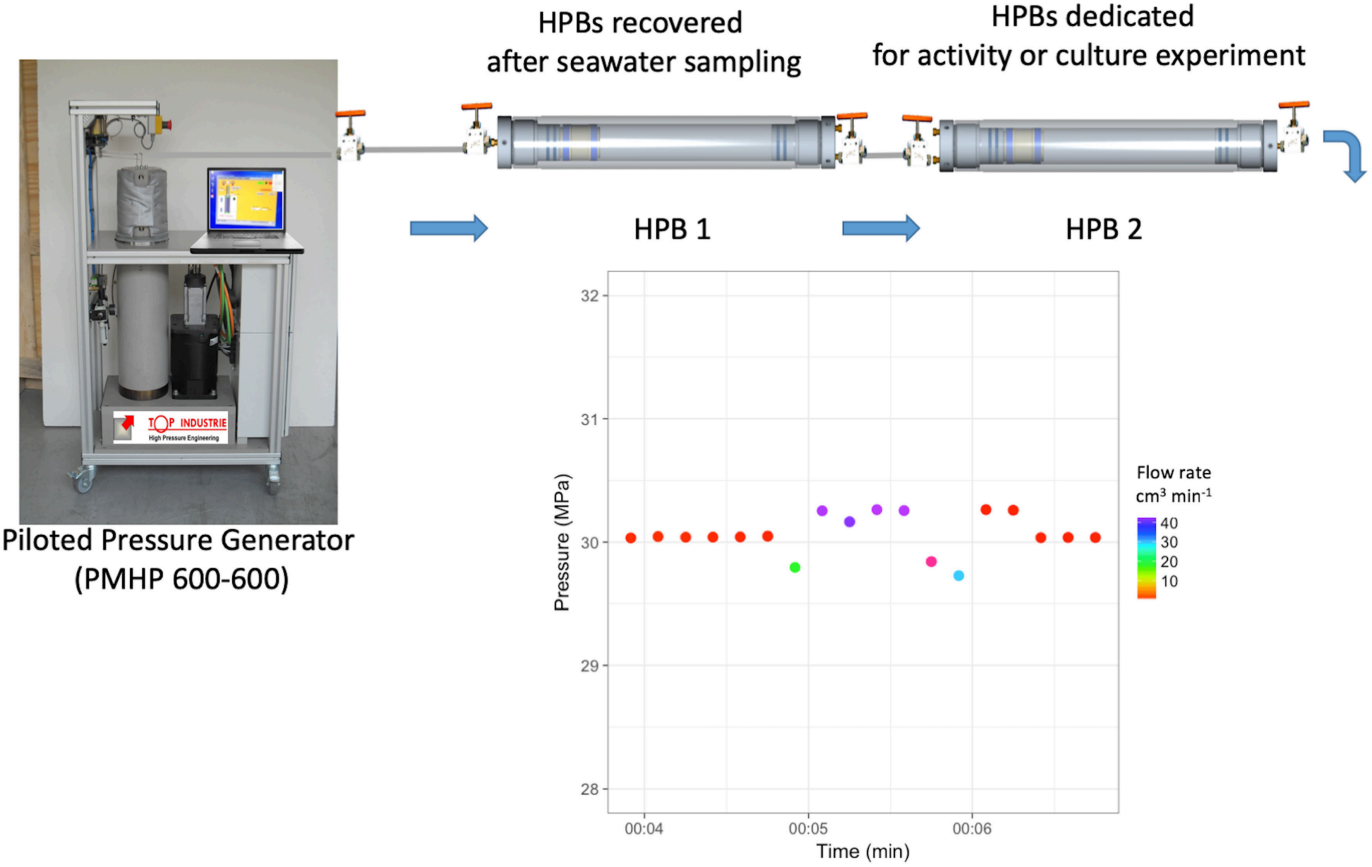
Transfer in equi-pressure mode. After sampling HPBs dedicated to sample (HPB 1) are connected at bottom end-cap to piloted pressure generator (PPG) (PMHP 600-600) and at top end-cap to HPB dedicated to activity measurement or culture experiments (HPB 2), previously pressurized at the working pressure. In the example, the PMHP 600-600 and the HPB2 are preconditioned to 30 MPa, the same hydrostatic pressure of the 3,000 m-depth sample of the HPB1. During the transfer, the PMHP 600-600 prevents pressure loss by adding milliQ water at the working pressure into the lower part of HPB 1, the sample being isolated by the floating-piston. The equivalent volume of the seawater sample enters HPB 2, which is managed by a handle valve at the bottom end-cap. Data is recorded during transfer in equi-pressure mode with a time-step of 10 s. The transfer takes about 2 min for a volume of 40 mL with an accuracy of <0.5 MPa.

Figure 3 describes the set-up used to transfer under equi-pressure mode. It is composed of three main parts: the PMHP 600-600, the 500 mL-HPB containing seawater sample (entitled thereafter HPB 1) and the HPB with a labeled tracer to measure activity or HPB with a culture medium (entitled thereafter HPB 2). To facilitate handling and to reduce experiment costs, 50 mL HPBs (2730-4000, Top Industrie SA) may be used instead of 500 mL HPBs. Both volumes of HPB can be used to perform a transfer in equi-pressure mode if the HPBs are equipped with a floating-piston inside.

Firstly, HPB 2 is filled with the labeled tracer or the culture medium and pre-conditioned at the *in situ* temperature. This operation is done at atmospheric pressure using a precision syringe in sterile conditions. Then, the HPB 2 is pressurized at the same pressure as the HPB 1 to equilibrate the pressure between both HPBs. Finally, both hand valves of the HPBs (at the top) are connected using a sterile 1/8″ (3.2 mm O.D.) stainless tube. The bottom part of the HPB 1 is connected to the PMHP 600-600. The top hand-valve of HPB 1 is opened to equilibrate the pressure with the small stainless-tube connection. The top hand-valve of HPB2 can be opened. The bottom part of the HPB 2, also fitted with a 1/8″ hand valve, is then slowly opened by the operator to create a weak leak, the PMHP 600-600 compensates the pressure loss by an increase in the flow rate. The PMHP 600-600 regulates the hydrostatic pressure by injecting distilled water into the bottom part of the HPB1, separated from the original deep-sea sample by the floating-piston. The operator regulates the volume using the end hand-valve. The PMHP 600-600 regulates the hydrostatic pressure in both HPBs, while the operator manages the transferred volume. Hence, the original deep-sea sample is transferred from HPB 1 into HPB 2 at constant pressure. HPBs are covered with a survival blanket to limit temperature change. From 500 mL samples, it is possible to transfer sub-samples of different volumes to perform replicates for metabolic activities or culture experiments under *in situ* pressure conditions. Less than two minutes is required to transfer 40 mL of sub-sample with a pressure accuracy of < 0.5 MPa (see the graph in Figure 3 and animated diagram in Supplementary Video 2).

### Example of Field Application

#### *In situ* Sampling and Incubation Experiment Under High-Hydrostatic Pressure

**Figure 4:**
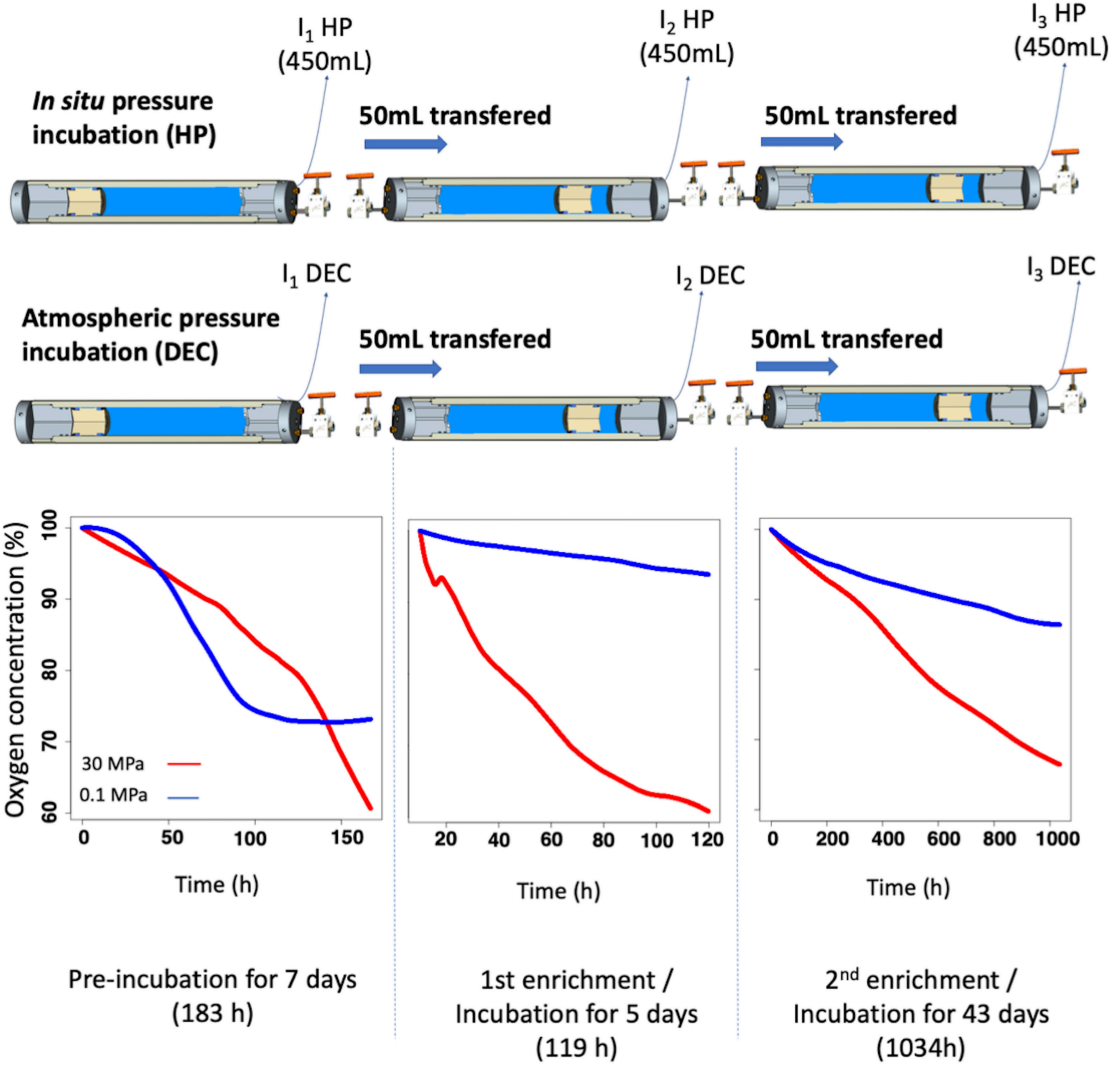
Timeline of the incubation experiment. Deep-sea samples were recovered at 3,000 m-depth and incubated at *in situ* pressure conditions (HP) or after decompression at atmospheric pressure conditions (DEC). Samples were firstly incubated at *in situ* temperature over 7 days. Then, the second incubation was made by transferring, in equi-pressure mode for each condition, 50 mL of the first incubated sample into a new high-pressure bottle (HPB) containing 450 mL of 0.2 μm-filtrated and sterilized seawater, sampled at the same depth. The remaining 450 mL of the first-incubated HPBs were then quickly sampled and conditioned for sequencing analysis (T_0_ HP and T_0_ DEC). At the end of the 2nd incubation, a third incubation was made according to the same protocol. At the end of each incubation the remaining 450 mL was conditioned for sequencing analysis and named T_1_ HP/T_1_ DEC and T_2_ HP/ T_2_ DEC, respectively. Dissolved-oxygen concentration was used to determine the incubation time. Graph plots represent dissolved-oxygen concentration (expressed in percent of initial dissolved-oxygen concentration) against time (h).

The experiment was conducted during the PEACETIME cruise (http://peacetime-project.org/) in May 2017 in the Ionian Sea. Seawater was sampled from 3,000 m-depth (Station ION, 35.49N, 19.78E) using two HPSUs, one under *in situ* conditions (30 MPa) and another one decompressed during up-cast (HPSU mounted without check valve) and incubated at atmospheric pressure (0.1 MPa). Details of the PEACETIME cruise will be fully described in a special issue soon. Incubations were carried out at *in situ* temperature (i.e., at 13°C in the deep Mediterranean Sea) for 7 days. At the end of this first incubation (I_1_ in Figure 4), fifty milliliters (50 mL) were transferred in equi-pressure mode into another HPB previously filled with 450 mL 0.2-μm-pore size filtered-seawater (to remove particulate matter and microorganisms), obtained at the same time and at the same depth using regular Niskin bottles for the second incubation (I_2_). The remaining volume was filtered, in <15 min, through 0.2-μm-pore-size filters (Millipore®, GPWP04700) without any fixation and stored at −80°C for DNA/RNA metabarcoding analysis. A third incubation (I_3_) is performed with the same protocol. A total of three incubations were performed maintaining *in situ* pressure (HP) or decompressed and incubated at atmospheric pressure conditions (DEC) (see workflow in Figure 4). The concentration of dissolved-oxygen was monitored during the incubation using optode sensors (see section *In situ* oxygen consumption and Figure 4).

#### Prokaryotic Heterotrophic Production

Prokaryotic heterotrophic production (PHP) was measured by incorporating L-[4,5-^3^H]-Leucine, (^3^H-Leu, 109 Ci mmol^−1^ of specific activity, PerkinElmer®) to get a final concentration of 10 nM in HPBs. Saturation concentrations were previously defined by multi-concentration kinetic experiments at atmospheric pressure (data not shown) and according to previous experiments (e.g., Tamburini et al., 2002). Transfer and sub-sampling of high-pressure samples were performed using a piloted pressure generator to ensure that hydrostatic pressure was maintained throughout the procedure within the mobile MIO-HPLab container (certified for the use of radiolabeled compounds). Figure 3 and Supplementary Video 2 present an example of the transfer from the HPB containing the 3,000 m-samples, obtained with the HPSU (HPB1), and the HPB pre-filled with the ^3^H-Leu (HPB2). In this case, forty millimeters of deep-sea samples were transferred into 3 independent HPBs (triplicate), amended with ^3^H-Leu, for samples maintained at *in situ* pressure conditions (HP). In parallel, triplicate 40 mL formaldehyde-killed blanks and triplicate 40 mL decompressed atmospheric pressure (DEC) samples were incubated with a final concentration of 10 nM of ^3^H-Leu. All the replicates (3 HP, 3 DEC, 3 blanks) were incubated at *in situ* temperature (13°C) in a Peltier-cooled incubator (Memmert® IPP750 plus). After 10 h of incubation, samples were fixed with 2% final concentration formaldehyde and stored at 4°C until filtration. The following protocol is detailed in Kirchamn (1993). To calculate the PHP, we used the empirical conversion factor of 1.55 ng C pmol^−1^ of incorporated ^3^H-Leu according to Simon and Azam (1989) assuming that isotope dilution was negligible under these saturating concentrations. The concentration of ^3^H-Leu within HPBs was checked at the end of the experiment based on 100 μL of samples.

#### *In situ* Oxygen Consumption

To measure, dissolved-oxygen consumption inside HPBs, we modified the top end-cap of the HPB with a sapphire window for optical measurement (see details in “High-pressure bottles” section and Supplementary Image 2). We have adapted an available solution based on an optical method using a non-invasive planar optode method which provides high-frequency measurements. The optical oxygen-sensor spot was glued with silicon glue onto the sapphire window directly inside the HPB rather than using a flow-through cell as in the pressure incubation system of McNichol et al. (2016). The oxygen sensor, containing the photoluminescent quenching dye, is produced by Presens GmbH® (Pst3, detection limit 15 ppb, ≈ 0.47 μM) or by PyroScience GmbH® (OXSP5, detection limit 0.3 μM). On the other side of the sapphire window, a polymeric optic-fiber was held against the sapphire window and was connected to a data logger. We used OXY-10 mini device for Presens GmbH® or FireStingO2 (fiber-optic oxygen meter) coupled to TeX4 (temperature extension module) for PyroScience GmbH®. Both data loggers shone a beam of light at a precise wavelength (for Presens 485 nm/620 nm, for PyroScience 620 nm/760 nm) onto the optode spot from the outside. Oxygen concentration data were then collected every minute during the incubation. Particular attention must be given to maintaining a constant temperature (here the samples were maintained at 13 ± 0.1°C) during the whole incubation to avoid a temperature sensor dependence signal during the experiment. The optodes were calibrated manually using a two-point calibration procedure. All optodes are intercalibrated individually and intercompared. Hydrostatic pressure, temperature and salinity are compensated for using algorithms proposed by McNeil and D'Asaro (2014) and García et al. (1992). In the Figure 4, dissolved-oxygen concentration is expressed as a percentage of initial concentration to normalize the differences in absolute concentration at the initial time of each incubation.

#### DNA and RNA Extraction and PCR Amplification

The 16S-rDNA (revealing all taxa present in a given sample) and 16S-rRNA (revealing taxa actively transcribing RNA) were taken from the same filter. 16S-rDNA and 16S-rRNA were extracted on two different pieces of filter. Each piece of filter was treated with TE-Lysis buffer (20 mM Tris, 25 mM EDTA, 1 μg μL^−1^ Lysozyme) followed by 10% SDS. The extractions were performed twice with an equal volume of phenol:chloroform:isoamyl alcohol pH8 16S-rDNA and pH 6 16S-rRNA. Then 16S-rRNA samples were treated with TurboDNase^TM^ (Ambion®, Thermo Fisher Scientific Corp.) and reverse transcribed into cDNA by RT-PCR using SuperScript® IV Reverse Transcriptase with random primers (Life Technologies, Thermo Fisher Scientific Corp.). For ribosomal diversity analysis, the V4 region of the bacterial and archaeal 16S rDNA genes and 16S cDNA product were amplified using universal primer sets (Caporaso et al., 2012), 515F-Y (5′ -GTGYCAGCMGCCGCGGTAA-3′, Parada et al., 2016) and 806RB (5′-GGACTACNVGGGTWTCTAAT-3′, Apprill et al., 2015) and using 2.5 U/50 μL TaKaRa PrimeSTAR® GXL DNA polymerase (OZYME). The 16S amplicons were sequenced by the MiSeq Illumina (paired end 2^*^ 250) platform GeT of Genotoul (https://get.genotoul.fr/en/). 16S-rDNA and 16S-rRNA raw reads sequences are deposited on public database GeneBank (accession number are ranged between SRR8503024 to SRR8503035).

#### 16S rDNA/rRNA Sequences Analysis

The paired-end raw reads were firstly overlapped and merged by platform GeT of Genotoul. The analysis of the 16S-assembled data relied on the use of QIIME 1.91 (Kuczynski et al., 2012). The removal of low quality bases and chimera sequences were performed by QIIME 1.91 script and UCHIME (Edgar et al., 2011), respectively. The high-quality sequences were then clustered into the Operational Taxonomic Unit (OTU) using the UCLUST algorithm and a threshold of 97% of sequence identity. The taxonomic assignment of representative sequences of OTUs was performed using the SILVA123 database (Edgar, 2010; Quast et al., 2013). The OTU table was filtered for low abundance OTUs (Bokulich et al., 2013). Finally, sub-sampling normalization, alpha and beta diversity were characterized by Phyloseq, an open-source software package project for R (www.r-project.org) (R Core Team, 2008; McMurdie and Holmes, 2013).

## Results and Discussion

In this article, we describe a field application using the pressure-retaining sampler and high-pressure systems that we developed. Samples taken from 3,000 m-depth were obtained in the Mediterranean Sea during the multidisciplinary cruise PEACETIME in May 2017. Three HPSUs were implemented on the CTD-carousel used during the PEACETIME cruise, one HPSU dedicated to the measurement of PHP and the other two for the incubation experiment described in Figure 4.

### Prokaryotic Activity Measurements

The prokaryotic heterotrophic production (PHP) measurements were performed to characterize the prokaryotic community lifestyle status with respect to hydrostatic pressure (Tamburini et al., 2013). PHP was 3.2-fold higher for samples maintained at *in situ* (PHP_HP_ = 0.93 ± 0.13 ng C L^−1^, *n* = 3) than those decompressed and incubated at atmospheric pressure conditions (PHP_DEC_ = 0.29 ± 0.01 ng C L^−1^, *n* = 3). Thus, the natural 3,000 m-depth prokaryotic assemblage sampled can be defined globally as piezophilic (that means adapted to *in situ* high pressure conditions) according to Tamburini et al. (2013) and Wannicke et al. (2015). This global adaptation of the prokaryotic assemblage to pressure is supported by the prokaryotic respiration rate (PR) measurements derived from oxygen consumption rates within the two HPBs during the incubation experiment. One set of samples was maintained at *in situ* hydrostatic pressure (HP) and the other was decompressed and incubated at atmospheric pressure (DEC). Table 1 and plots presented in Figure 4 summarize the O_2_-consumption results. Oxygen consumption is clearly always higher in HP than in DEC conditions as exemplified by the third incubation experiment where only 14% of the O_2_ is consumed in DEC conditions compared to 34% in HP conditions after 43 days of incubation (see O_2_ graph-plots in Figure 4). Results in Table 1 show that for each incubation experiment, PR was between 1.6 and 7.0-folds higher under *in situ* pressure conditions than in incubations carried out after decompression and incubated in atmospheric conditions. Furthermore, the sampling strategy of the incubation experiments was adjusted using real-time monitoring of oxygen concentrations. All incubation experiments were performed until oxygen consumption reached a maximum of 40%.

**Table 1:** Total cells count (stained with DAPI), range of prokaryotic respiration, number of observed operational taxonomic units (OTUs), 16S-rDNA- and 16S-rRNA-based sequence lengths, and alpha-diversity indexes for enrichment incubation experiments (Figure 4).

	**I_**1**__HP**	**I_**1**__DEC**	**I_**2**__HP**	**I_**2**__DEC**	**I_**3**__HP**	**I_**3**__DEC**
DAPI cells count 10^5^ mL^−1^	2.05 ± 0.33	1.97 ± 0.29	2.39 ± 0.36	1.73 ± 0.25	6.78 ± 0.79	2.01 ± 0.32
Prokaryotic respiration (μmol O_2_ L^−1^ h^−1^) (min-max)	0.55–2.5	0.15–1.15	0.32–2.39	0.09–0.22	0.07–0.15	0.01–0.09
Total number OTU 16S-rDNA-based	108	120	126	136	128	131
Total number OTU 16S-rRNA-based	129	110	139	148	109	155
Means length of sequence DNA (bp)	305.2 ± 31.3	300.2 ± 25.3	297.1 ± 20.9	299.8 ± 25.0	299.8 ± 25.0	308.0 ± 33.7
Means length of sequence RNA (bp)	300.0 ± 25.7	300.2 ± 25.3	300.0 ± 25.5	299.8 ± 25.0	302.4 ± 28.6	309.2 ± 34.5
Simpson DNA	0.77	0.40	0.77	0.85	0.90	0.87
Simpson Rarefied DNA	0.77	0.40	0.78	0.84	0.90	0.87
Simpson RNA	0.83	0.23	0.81	0.81	0.90	0.86
Simpson rarefied RNA	0.83	0.24	0.81	0.82	0.90	0.87

### Prokaryotic Diversity and Community Structure (16S rDNA and rRNA)

The prokaryotic diversity and community structure shifts were characterized by 16S rDNA (the resident community) and 16S rRNA (the active community) gene sequencing according to experimental conditions (HP and DEC). Beyond RNA instability and bias originating from DNA/RNA extraction and amplification, the rRNA- overcomes rDNA-based approach for detecting live prokaryotic cells in water (Li et al., 2017).

**Figure 5:**
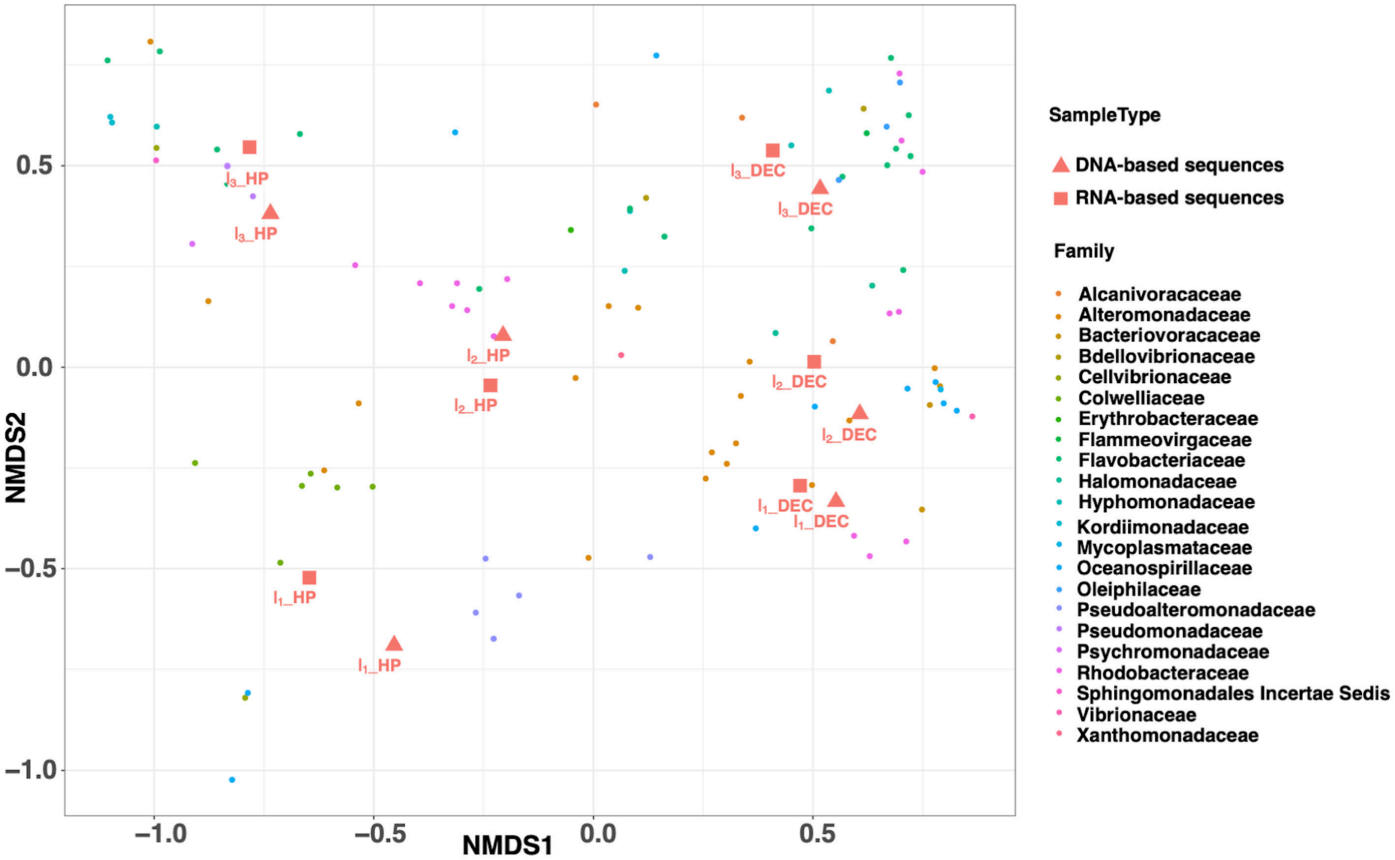
NMDS ordination plot (Bray–Curtis distance matrix) from incubation experiment performed with deep-seawater sample maintained at *in situ* condition (HP) and after decompression and incubated at atmospheric pressure (DEC). This graph shows two clusters: on one side the HP samples and on the other the DEC samples. Prefix D and R before the name for the sample is for DNA- or RNA-based sequences, respectively. The NMDS stress is equal to 0.03.

The amplicon sequencing followed by trimming and normalization processes generated 19420 16S rDNA and 16S rRNA high quality reads (average length 302 bp) per sample (Table 1). In addition, the number of observed OTUs ranged from 108 to 155 over time with no significant difference between rDNA and rRNA analysis. The decrease of the specific richness during the experiment was indicated by the Simpson index, which increases from 0.77 to 0.90 and from 0.40 to 0.87 within HP and DEC samples, respectively. Moreover, the comparison of the OTUs relative abundances shared between rDNA and rRNA [log(rDNA/rRNA)] showed a distribution around the 1:1 bar, especially for the more abundant ones (Supplementary Image 4). Beyond the pitfalls linked to the overestimation or underestimation of OTUs abundances, this result suggests that the active community corresponded to almost the entire resident community. This finding is fully supported by a non-metric multidimensional scaling (NMDS) analysis that highlighted close positions for rDNA and rRNA samples with common experimental conditions (Figure 5). In contrast, the shifts observed in prokaryotic community structure over time are driven by the different experimental conditions, confirming a previous study (Wannicke et al., 2015). Indeed, HP vs. DEC incubation conditions were mainly discriminated by the absence of *Colwelliacea* and *Bacteriovracaceae* in DEC incubations (NMDS1 axis). This is already the case at the end of the 1st incubation (I_1__HP vs. I_1__DEC) and reinforces the results that decompression impacts not only prokaryotic activity as shown in this study and elsewhere (Tamburini et al., 2013; Wannicke et al., 2015) but also affects the community composition (La Cono et al., 2009, 2015) or the gene expression (Edgcomb et al., 2016).

**Figure 6:**
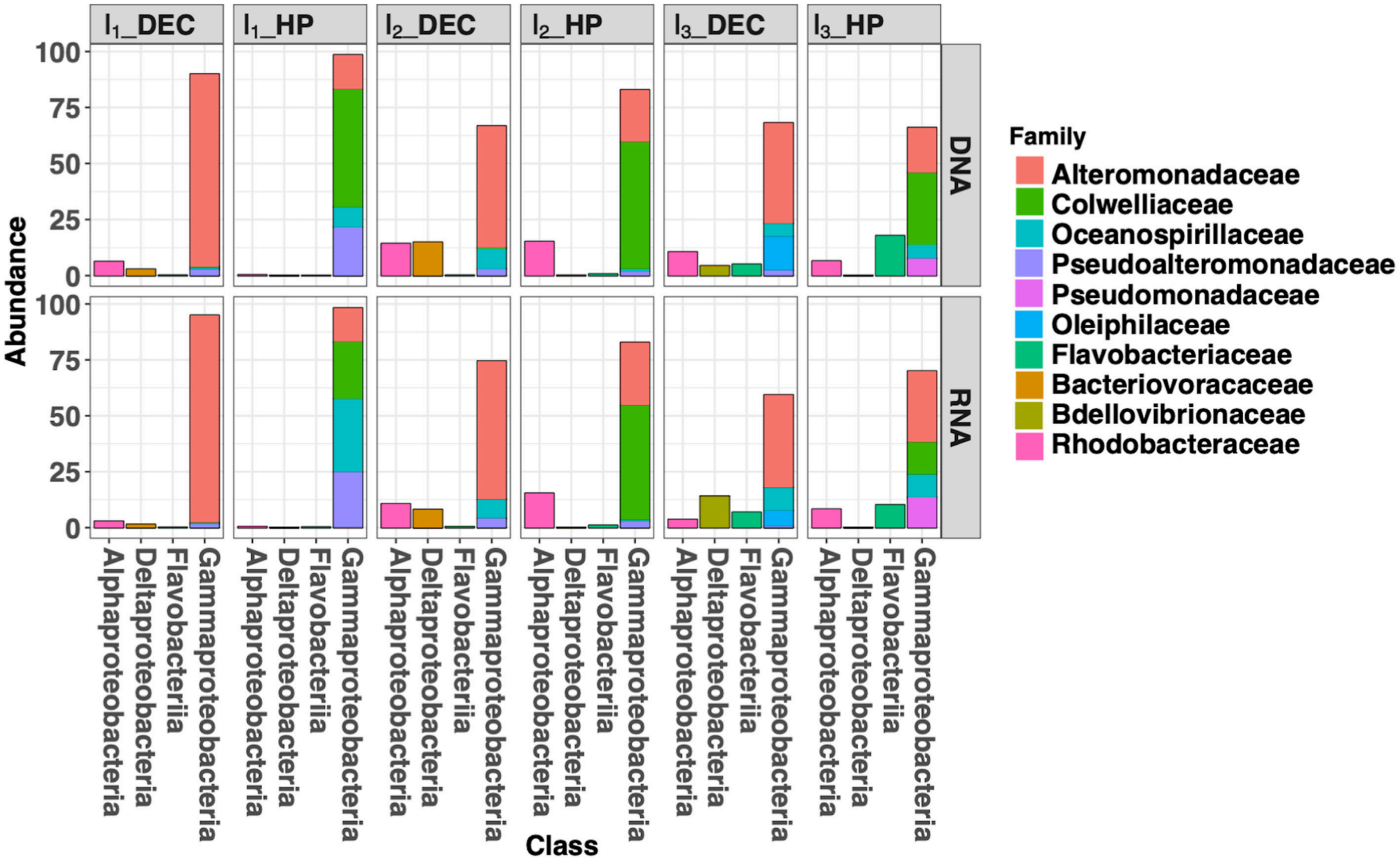
Bar plot of the 10 most abundant families into the four main classes during incubation experiments of deep-seawater samples maintained at *in situ* pressure conditions (HP) and after decompression and incubated at atmospheric pressure conditions (DEC).

For each experimental condition (HP and DEC), variation in community structure over time (incubation time-series) was observed and driven by the NMDS2 axis (Figure 5). This suggests that sufficient carbon and energy is available in the deep-sea water sampled at 3,000 m-depth used as the “medium” in our incubation experiments. Figure 6 shows the shifts in prokaryotic community structure from the ten most abundant families (corresponding to four classes) during the course of the incubation experiments. After the 1^st^ incubation experiment (I_1_) no *Archaea* were detected in either condition whilst they can reach 50% of the prokaryotic community in the deep ocean (Karner et al., 2001; Herndl et al., 2005; Tamburini et al., 2009a). The absence of *Archaea* genes sequenced in the incubation experiment is confirmed by the RT-qPCR quantification showing more than 4-orders of magnitude between the number of *Archaea* and *Bacteria* gene copies per milliliter (data not shown). In DEC conditions (I_1__DEC), the class *Gammaproteobacteria* accounted for 93% of the total sequences and was almost exclusively represented by the family *Alteromonadaceae* (Figure 6). Conversely, in HP conditions (I_1__HP), the prokaryotic community was also dominated by the class *Gammaproteobacteria* (99% of the total sequences) but included various families such as *Alteromonadaceae, Oceanospirillaceae, Pseudoalteromonas*, and *Colwelliaceae*. The family *Alteromonadaceae* accounted only for 15% (of both 16S rDNA- and rRNA sequences), while *Colwelliaceae* dominated with 53 and 25% of 16S rDNA- and rRNA sequences, respectively. In our study, the family *Colwelliaceae* was mainly made up of the genus *Colwellia* (more than 50%) which contained isolates described as piezophilic (Deming et al., 1988; Nogi et al., 2004; Eloe et al., 2011). The proportions of family *Pseudoaltermonadaceae* reached 22 and 25% of 16S rDNA- and rRNA sequences, respectively. Interestingly, *Oceanospirillaceae* represented 9% of 16S rDNA sequences but 33% of 16S rRNA sequences at I_1__HP. The family *Oceanospirillaceae* is one of the rare families containing piezophilic isolates (Cao et al., 2014).

At the end of the third incubation experiment (I_3_, Figure 4), the active community (rRNA) of the *Gammaproteobacteria* decreased around 60 and 70% in DEC and HP conditions, respectively. Under DEC conditions, this decrease was in favor of *Bdellovibrionaceae* (family *Deltaproteobacteria*) which is mainly represented by the genus OM27, previously described as bacterial predatory (Fuchs et al., 2005; Orsi et al., 2016) and was only found in the DEC condition. This result suggests that high hydrostatic pressure could initiate growth repression of these microorganisms within this family. In contrast, at I_3__HP, an increase in the abundance of families *Rhodobacteraceae* (*Alphaproteobacteria* class) and *Flavobacteriaceae* (*Flavobateria* class) was observed and reached 10 and 8% of the rRNA total sequences, respectively. Both of these families were absent from the “active community” at I_1__HP. The family *Rhodobacteraceae* was found in both DEC and HP conditions in proportions ranging from 6 to 15% and are ubiquitous from the surface to the deep ocean (Moran et al., 2003). Members of the family *Flavobacteriaceae* have shown the ability to degrade high molecular dissolved organic matter (HMW-DOM) such as chitin, agar or particulate organic matter (POM) that characterize the deep-sea environment (Kirchman, 2002; Fernández-Gómez et al., 2013). It is widely known that the deep-sea waters are depleted in carbon and energy sources. Consequently, deep-sea microorganisms harbored unique metabolic capabilities with respect to the degradation of complex organic matter and were supported by genomic and transcriptomic analysis (Vezzi et al., 2005; Delong et al., 2006) as well as the measurements of degradation of refractory organic matter in deep water compared with surface water (Hoppe and Ullrich, 1999; Teira et al., 2006; Tamburini et al., 2009a; Boutrif et al., 2011).

## Conclusion and Perspectives

Conserving the *in situ* conditions when sampling the deep ocean is becoming a major concern for the scientific community and many have tried to find solutions to sample without decompressing the samples. For example, Shillito and collaborators developed PERISCOP to sample deep-sea macro-organisms (e.g., shrimps, crabs), under *in situ* conditions without decompression issues. This was then adapted by BALIST into IPOCAMP for use in physiology and behavioral experiments (Shillito et al., 2008, 2014; Ravaux et al., 2013). Likewise, McNichol et al. (2016) developed a specific device to sample hydrothermal vent fluids under *in situ* conditions and to study microbial metabolism associated with fluid biogeochemistry. Peoples et al. (2019) proposed a pressure-retaining sampler (coupled to a Lander) capable of collecting hadal seawaters under *in situ* conditions during recovery. Finally, Parkes et al. (2009) have proposed high-pressure sampler systems to study prokaryotic subseafloor sediments.

Based on the prototype proposed by Bianchi et al. (1999) and our experience with more than 200 deep-water samples collected under *in situ* pressure conditions, we propose a 6,000 m-seawater pressure-retaining sampler ready-to-use for the scientific community. The various improvements implemented promote the success of high-pressure sampling, sub-sampling, and transfer of samples in equi-pressure mode in replicate. In addition, it is also possible to directly measure oxygen consumption at high hydrostatic pressure and to use radiolabeled compounds in accordance with health and safety standards. Such methodology can be an outstanding tool in isolating new piezophilic strains without any pressure losses during the experiment.

Apart from collecting samples and maintaining them under *in situ* conditions, it is important to note that this system and specifically HPBs are versatile. For example, 316L stainless-steel with PEEK coating HPBs can be selected for cultivation under anoxic conditions (e.g., sulfato-reducing bacteria) they don't corrode. HPBs and PPGs can be used to perform laboratory experiments such as for sinking particle simulation experiments (Tamburini et al., 2006, 2009b; Riou et al., 2017). To our knowledge, several teams already have the same pressure-retaining sampler (Deep Carbon Observatory, DCO—Sloan Foundation; Hadal Science and Technology Research Center, HAST, Shangai, China; Sanya Institute of Deep Sea Science and Engineering, SIDSSE, China Academy of Science). Using the same HPBs, IBIS is being developed by IFREMER for the sampling of hydrothermal fluids.

The incubation experiment, presented in this study, is an example of a field application using the pressure-retaining sampler and associated high-pressure systems. This illustrates its fully operational use during our field experiment dedicated to deep-sea microbial oceanography. Using prokaryotic diversity and community structure based on 16S rDNA- and rRNA-sequences, we have shown that the diversity decreased in both HP and DEC conditions and that the community structure evolved differently according the pressure conditions. Further studies will be conducted to reveal the metabolic pathways and microbial taxa involved in the biogeochemical transformation of the organic matter in the dark ocean, as many important ecological and biogeochemical processes, linked to the biological carbon pump, take place in this the largest habitat of the biosphere.

## Author Contributions

MG and CT wrote the initial draft and coordinated the drafting of the paper, and participated in the experimental design. The high-pressure experiments were carried out by MG, CT, MR, and NB. SG and PB participated in molecular biological work. MG and FA performed sequencing analyses. All authors contributed to the discussion and the writing of the paper.

### Conflict of Interest Statement

The authors declare that the research was conducted in the absence of any commercial or financial relationships that could be construed as a potential conflict of interest.

## Supplementary Material

Supplementary Image 1 Top end cap with optic measurement. (1) Optic fiber, (2) Temperature sensor (pt100), (3) Inlet/outlet 1/8″ diameter (3.2 mm O.D.), (4) O'ring, (5) Planar optode (diameter 5 mm), (6) Sapphire window, (7) Titanium screw. This modified top end-cap can be fitted indifferently on TA6V HPBs or on 316L stainless-steel with PEEK coating HPBs. However, the latter has to be pre-conditioned to equilibrate O_2_ concentration with PEEK coating. In this experiment, we have used TA6V HPBs to follow the oxygen concentration during the experiments both at atmospheric and *in situ* pressure conditions.

Supplementary Image 2 Schematic drawing of sampling valve fitted on High-Pressure Sampler Unit (HSU). 1. Nylon wire; 2. Stainless-steel trigger; 3. Stainless-steel frame for valve; 4. Modified swagelock valve (réf: SS-84PS4); 5. Modified handle to link trigger at spring; 6. sample inlet, 7. spring. In this view sampling valve is set in close position before sampling **(A)**. At the sampling depth nylon wire is disconnected to carrousel trigger, modified Swagelock® valve move to opening position performing a quarter of a rotation by power of the extended spring **(B)**.

Supplementary Image 3 Photograph showing different point of view of the mobile MIO-HPLab container. This mobile laboratory has been constructed in a 20 feet container. It is composed of two piloted pressure genrators (PPGs), four temperature regulated water baths with two temperature coolers dedicated for High-Pressure Sampler Unit (HPSU) and particle sinking experiments (PASS) experiments (Tamburini et al., 2009b), and a reinforced Peltier-cooled incubator Memmert IPP 750 oven for HPBs incubation. The mobile laboratory (MIO-HPLab container) is also certified to use radiolabeled during oceanographic cruises.

Supplementary Image 4 RNA:DNA ratios from RNA-based and DNA-based relative log transformed abundance of OTU. This ratio enables us to estimate a proportion of active prokaryote for each sample. The black line is 1:1 bar. OTU are distributed around the line which indicates that the microbial communities identified in the sample are also active.

Supplementary Video 1 Animated schematic drawing in transparency of the front and back views of the High-Pressure Sample Unit (HPSU). **(A)** During the descent of the CTD-carousel. The inlet-valve is in close position; **(B)** During the sampling at the chosen depth after firing the CTD-Carousel and the inlet-valve is in open position. The numbers are the same than in Figure 1: 7. exhaust tank; 8. Polypropylene main frame; 9. Push rod for attachment to a rosette system. When the (10) inlet-valve is opened by magnetically activated lanyard release, the seawater enters the hydraulic circuit through a 1/8″ (3.2 mm O.D.) stainless-steel tube (11), via one check valve (12), to fill in the HPB (13), the pressure accumulator (14) and the aero-hydraulic pressure sensor (15).

Supplementary Video 2 An animated diagram showing a transfer in equi-pressure mode between two High-Pressure Bottles (HPBs). HPB1 is the high-pressure bottle containing the 3,000 m-depth sample maintained under *in situ* pressure conditions. HPB2 is the high-pressure bottle containing radiolabeled compounds, such as, ^3^H-Leucine to measure prokaryotic heterotrophic production (PHP). Forty milliliters of 3000 m-depth seawater sample from HPB1 are transferred into HPB2 containing 10 nM of aqueous ^3^H-Leucine. This process is performed, securely, in replicate inside the mobile MIO-HPLab container.
