# Supplementary figures and images for: Pressure-Retaining Sampler and High-Pressure Systems to Study Deep-Sea Microbes Under in situ Conditions
Source: Front Microbiol. 2019 Apr 9;10:453. doi: 10.3389/fmicb.2019.00453 (PMC6465632; doi:10.3389/fmicb.2019.00453)

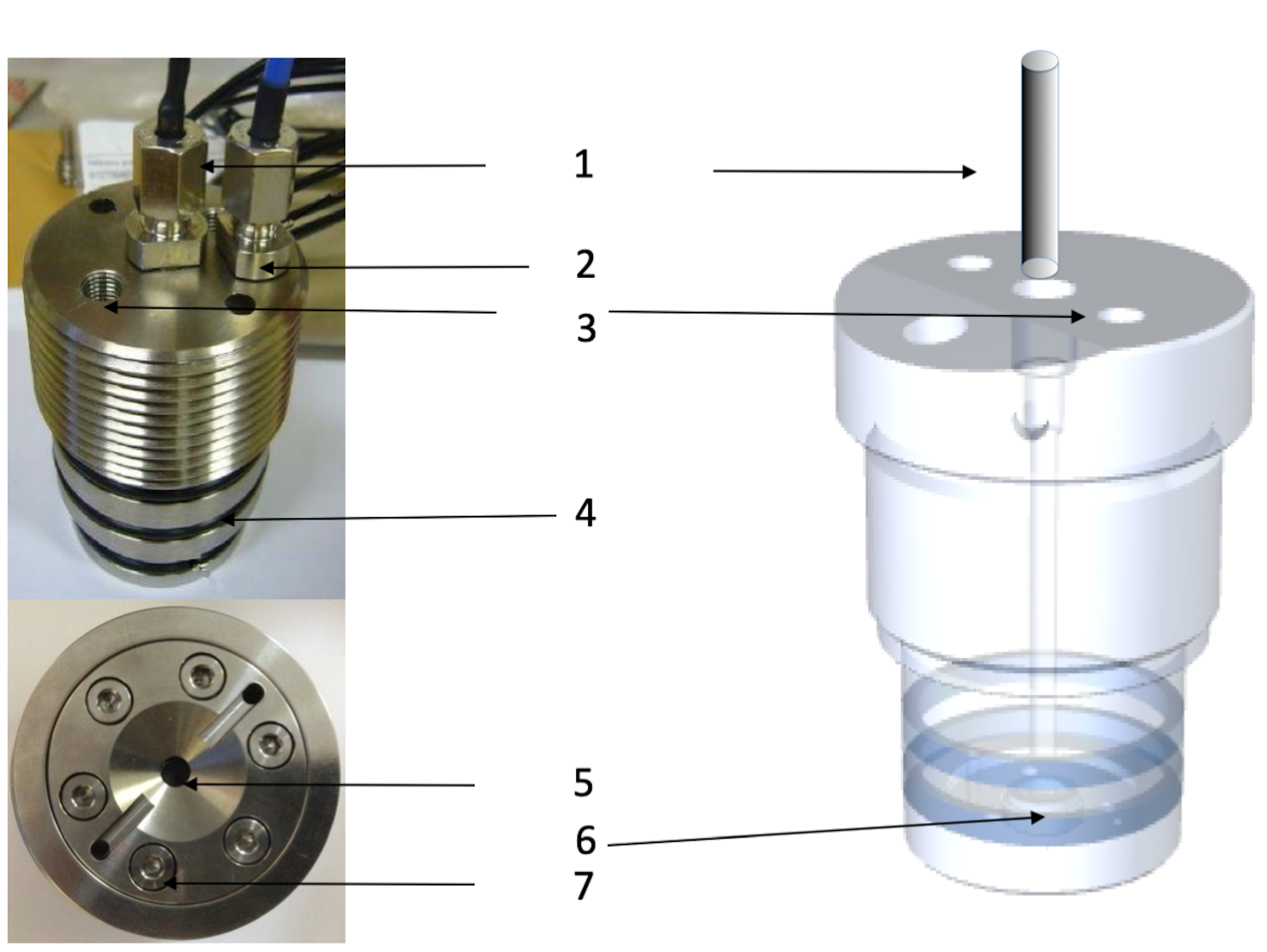

Supplement: Supplementary Image 1 — Top end cap with optic measurement. (1) Optic fiber, (2) Temperature sensor (pt100), (3) Inlet/outlet 1/8″ diameter (3.2 mm O.D.), (4) O'ring, (5) Planar optode (diameter 5 mm), (6) Sapphire window, (7) Titanium screw. This modified top end-cap can be fitted indifferently on TA6V HPBs or on 316L stainless-steel with PEEK coating HPBs. However, the latter has to be pre-conditioned to equilibrate O2 concentration with PEEK coating. In this experiment, we have used TA6V HPBs to follow the oxygen concentration during the experiments both at atmospheric and in situ pressure conditions. [file Image_1.TIFF]

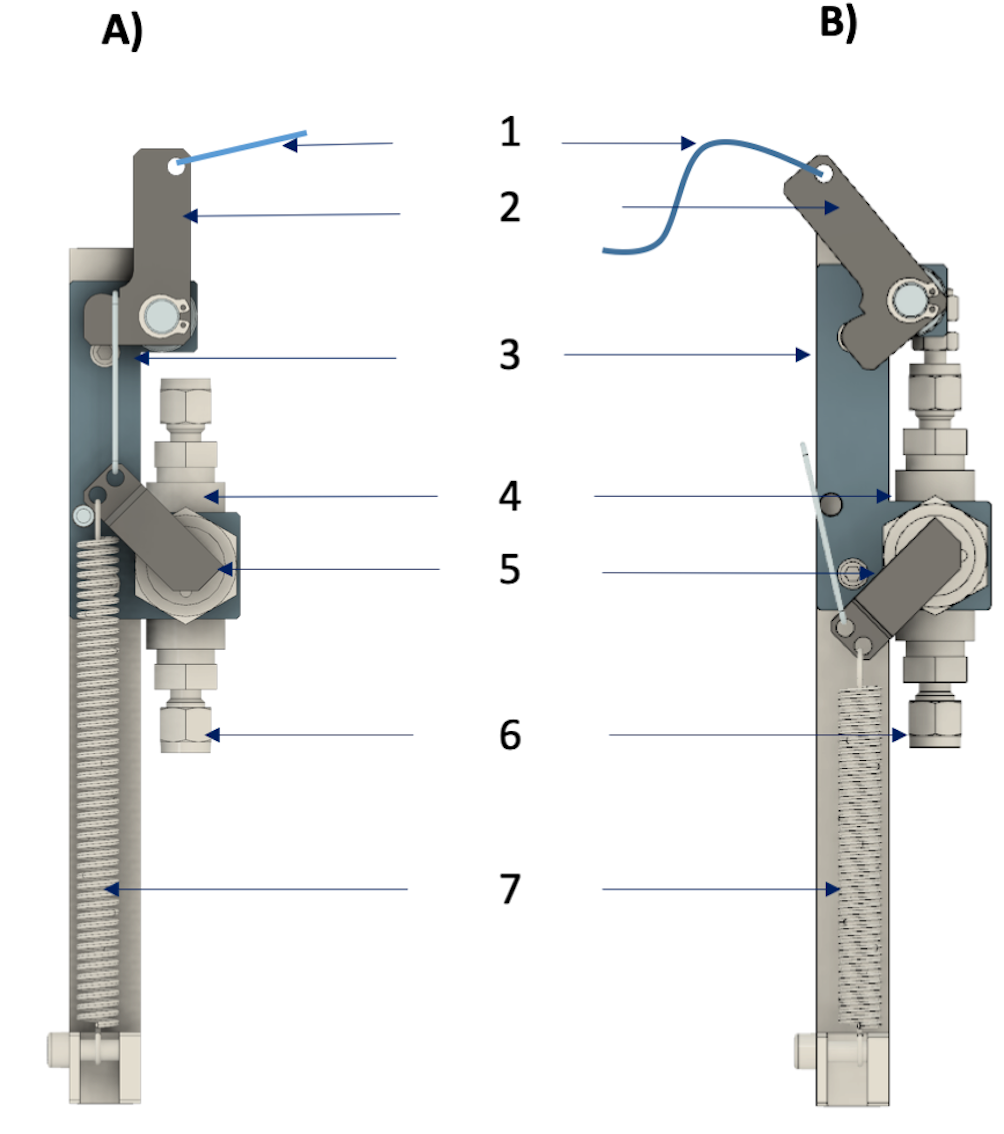

Supplement: Supplementary Image 2 — Schematic drawing of sampling valve fitted on High-Pressure Sampler Unit (HSU). 1. Nylon wire; 2. Stainless-steel trigger; 3. Stainless-steel frame for valve; 4. Modified swagelock valve (réf: SS-84PS4); 5. Modified handle to link trigger at spring; 6. sample inlet, 7. spring. In this view sampling valve is set in close position before sampling (A). At the sampling depth nylon wire is disconnected to carrousel trigger, modified Swagelock® valve move to opening position performing a quarter of a rotation by power of the extended spring (B). [file Image_2.TIFF]

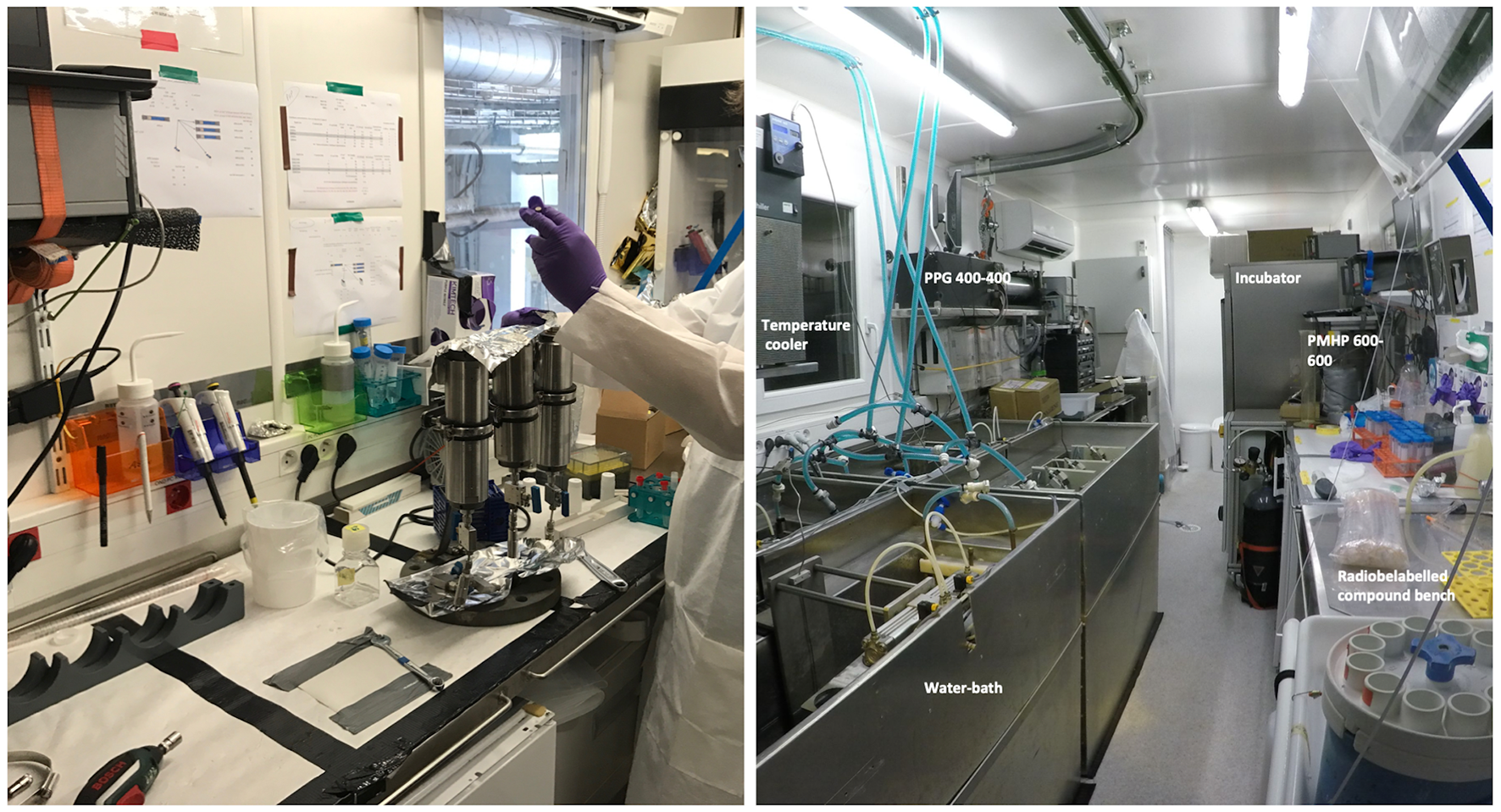

Supplement: Supplementary Image 3 — Photograph showing different point of view of the mobile MIO-HPLab container. This mobile laboratory has been constructed in a 20 feet container. It is composed of two piloted pressure genrators (PPGs), four temperature regulated water baths with two temperature coolers dedicated for High-Pressure Sampler Unit (HPSU) and particle sinking experiments (PASS) experiments (Tamburini et al., 2009b), and a reinforced Peltier-cooled incubator Memmert IPP 750 oven for HPBs incubation. The mobile laboratory (MIO-HPLab container) is also certified to use radiolabeled during oceanographic cruises. [file Image_3.TIFF]

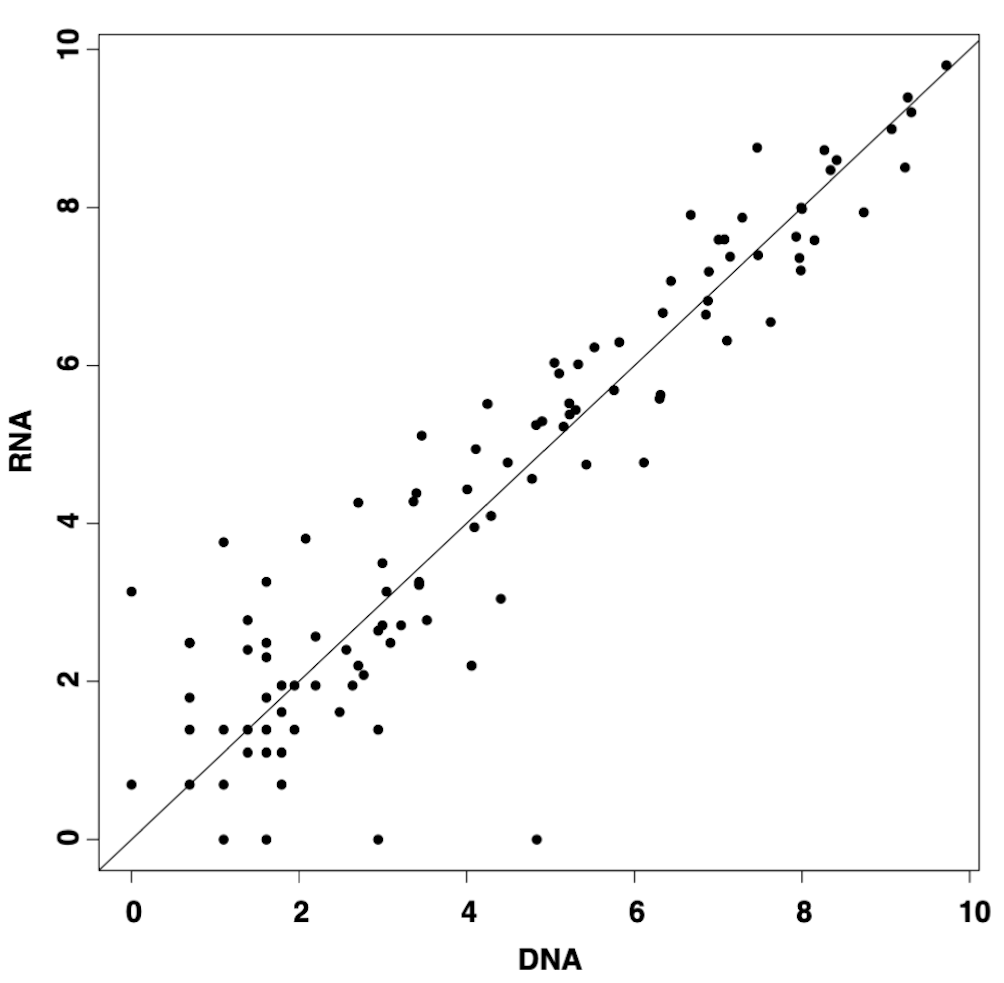

Supplement: Supplementary Image 4 — RNA:DNA ratios from RNA-based and DNA-based relative log transformed abundance of OTU. This ratio enables us to estimate a proportion of active prokaryote for each sample. The black line is 1:1 bar. OTU are distributed around the line which indicates that the microbial communities identified in the sample are also active. [file Image_4.TIFF]
